# Variable-specific preprocessing enables cross-scale transferable Raman models from high-throughput bioreactor data

**DOI:** 10.1186/s40643-026-01105-5

**Published:** 2026-08-29

**Authors:** Sung-Hyuk Han, Jiyun Park, Kwang-Bae Lee, Cheol-Hwan Park, Dong-Yup Lee

**Affiliations:** 1 Upstream Process Engineering, Manufacturing Science & Technology, R&D, GC Biopharma, 93 Ihyun-ro, 30beon-gil, Giheung-gu, Yongin, Gyeonggi-do 16924 Republic of Korea; 2 School of Chemical Engineering, Sungkyunkwan University, 2066 Seobu-ro, Jangan-gu, Suwon, Gyeonggi-do 16419 Republic of Korea

**Keywords:** Raman spectroscopy, Chemometric modeling, Process analytical technology (PAT), Cross-scale transferability, CHO cell culture, Data preprocessing

## Abstract

Raman spectroscopy is increasingly used as a process analytical technology (PAT) for real-time monitoring of bioprocesses. However, chemometric models developed using high-throughput (HT) mini-bioreactor systems often show limited predictive performance when applied to larger-scale processes, reflecting an out-of-distribution (OOD) challenge in cross-scale model transfer. In this study, we investigated whether variable-specific data preprocessing strategies can improve the cross-scale prediction performance of Raman chemometric models calibrated using HT cell culture data. Multiple preprocessing approaches were systematically evaluated for key cell culture target outputs, and the optimal preprocessing pipeline for each output was selected based on its ability to minimize cross-scale prediction error. The optimized variable-specific preprocessing pipelines reduced cross-scale prediction errors by 14.0–56.1% in RMSEP across the five initially poorly transferable target outputs compared with the standard single-scale preprocessing pipeline. Consequently, these optimized single-scale models achieved predictive accuracy approaching that of resource-intensive combined-scale models. Importantly, the improvement was not dependent on a specific preprocessing technique but rather on selecting preprocessing strategies suited to the spectral characteristics of each target output. These findings suggest that intelligent preprocessing selection can mitigate out-of-distribution effects in Raman chemometric models and enable more reliable prediction across scales using HT datasets for model calibration. This approach highlights the potential of Raman spectroscopy as a practical and scalable PAT tool for industrial bioprocess development.

**Graphical abstract:**

**Figure:**
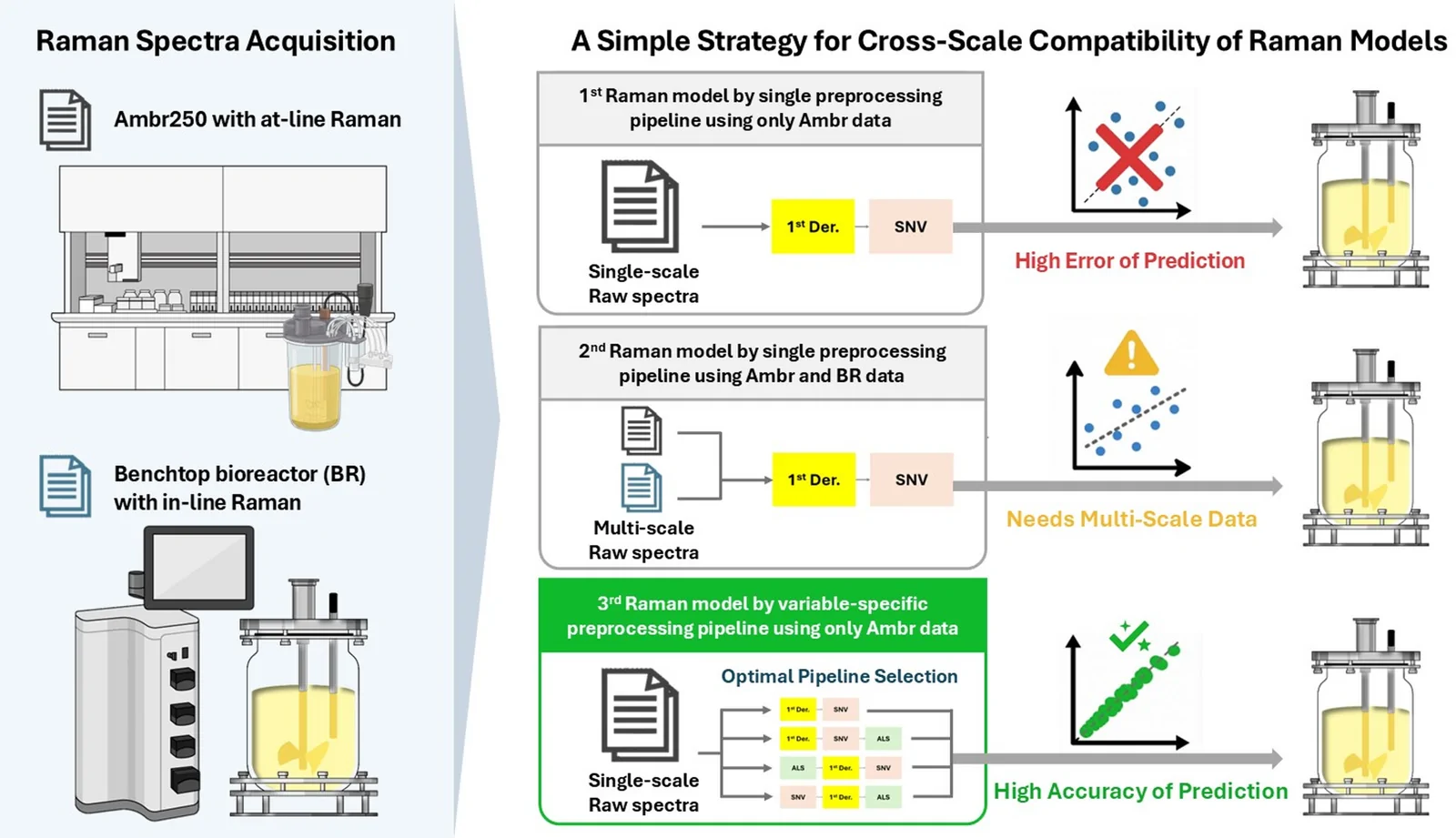

**Supplementary Information:**

The online version contains supplementary material available at https://doi.org/10.1186/s40643-026-01105-5.

## Introduction

 Biopharmaceutical manufacturing demands precise real-time monitoring and control to ensure product safety, efficacy, and consistent quality (Rathore and Winkle 2009). Biologics, including recombinant proteins and monoclonal antibodies, are structurally complex and therefore require tightly regulated environments for optimal expression and production (Esmonde-White et al. 2022). Because even minor variations in critical process parameters (CPPs) can substantially affect critical quality attributes (CQAs); robust monitoring, therefore, is essential (Han et al. 2025; McAndrew and Kauffman 2020). Process Analytical Technology (PAT) provides a framework for real-time monitoring that enhances process understanding and supports regulatory compliance (Adak et al. 2025; FDA 2004). Particularly during scale-up or site transitions, PAT tools are valuable for minimizing process variability (Esmonde-White et al. 2022). Among these tools, Raman spectroscopy has attracted growing interest for its ability to generate molecular fingerprints via inelastic light scattering. The technique operates in a non-destructive, label-free, and real-time manner, enabling simultaneous measurement of multiple process analytes and attributes directly in aqueous bioprocesses (Das and Agrawal 2011; Esmonde-White et al. 2017; Li et al. 2013; Rostron et al. 2016). In sensitive cell culture systems, Raman spectroscopy enables rapid detection of environmental changes and mitigates variability during process transfer (Dong et al. 2024; Rafferty et al. 2020).

To fully leverage Raman applications, chemometric models for key process analytes and attributes must be developed systematically. This process involves (1) data collection, (2) spectral preprocessing, and (3) regression model construction (Gibbons et al. 2023; Otto 2023). Because signals from target components are often weak or masked by noise, fluorescence backgrounds, and overlapping Raman bands from other matrix components, robust multivariate modeling is essential for accurate prediction (Dong et al. 2024; Otto 2023; Rafferty et al. 2020; Singh 2025). High-throughput systems, such as the Ambr^®^ 250 HT, have been increasingly adopted with at-line Raman spectroscopy to accelerate data collection and generate large, diverse datasets for model building while covering process variability through design of experiments (Sibley et al. 2020). However, a persistent challenge arises when models developed on these mini-scale systems lose predictive accuracy at larger scales (Rowland-Jones et al. 2021; Whelan et al. 2012). Poor cross-scale compatibility often results from cross-condition variability, in which differences in instrumentation, probe types, and spectral acquisition parameters between scales create substantial spectral variations unrelated to chemical composition (Pétillot et al. 2020). In practical bioprocess environments, scale transitions often inherently involve differences in instrumentation, optical probes, and spectral acquisition settings; therefore, cross-scale transfer in this study reflects a composite cross-condition challenge.

Various strategies have been investigated to overcome bottlenecks, largely focusing on either expanding the calibration dataset or developing alternative modeling techniques. Several studies have focused on integrating spectra acquired at multiple scales to develop combined-scale models (Berry et al. 2015; Lang et al. 2025). While robust, this approach requires extensive large-scale datasets, diminishing the efficiency of high-throughput workflows. Other approaches seek to build models from minimal data, such as using Indirect Hard Modeling (IHM) or synthetic data augmentation (Klaverdijk et al. 2025), or by constructing models from artificially mixed samples (Hara et al. 2023). Building on these data augmentation concepts, a recent study demonstrated the generation of synthetic spectral libraries (SSL) through the in silico spiking of pure component spectra into mammalian cell culture datasets, which enriches the spectral information content while considerably reducing the empirical cost and time required for model calibration (Hellequin et al. 2025). A third category explores technically driven methods, including advanced machine learning frameworks (Rashedi et al. 2025; Tulsyan et al. 2019) or instrument standardization (Pétillot et al. 2020). Formal model transfer approaches, such as calibration transfer (Feudale et al. 2002), instrument standardization (Pétillot et al. 2020), orthogonal signal correction (OSC) (Wold et al. 1998), domain adaptation (Pan and Yang 2009), and transfer component analysis (Pan et al. 2010), are widely used to address spectral differences across instruments, scales, or process domains. These methods aim to align spectral distributions, remove non-predictive variation, or learn domain-invariant latent structures, but often require target-domain data, transfer samples, or additional optimization.

In contrast, the present study does not propose a new transfer algorithm. Instead, it provides a complementary upstream strategy that systematically selects preprocessing methods according to the spectral characteristics of each target analyte or process attribute, thereby improving the quality of information entering the regression model prior to the application of formal transfer techniques.

Our contribution lies in demonstrating that systematic and variable-specific selection of existing preprocessing strategies can substantially improve cross-scale model transferability. To realize this, we introduce a methodology that focuses on the fundamental integrity of the intrinsic spectral data itself, rather than altering the regression algorithm or the data acquisition strategy. While standard baseline correction techniques, such as Asymmetric Least Squares (ALS), are widely accessible, their universal and uniform application across all target outputs often fails to account for the distinct spectral characteristics, such as concentration ranges and signal overlaps, of individual target analytes and process attributes. The key lies not in multi-scale datasets or reliance on a novel modeling technique, but in a systematic screening to select optimal, variable-specific preprocessing pipelines capable of isolating core chemical information from scale-dependent artifacts (e.g., background fluorescence). This addresses a critical gap in the literature: the absence of systematic, variable-specific evaluations of preprocessing as the primary enabler for single-scale model transferability, despite the recognized complexity and lack of standardization in this step (Barton et al. 2022; Gautam et al. 2015; Guo et al. 2021). To our knowledge, no previous studies have systematically demonstrated that cross-scale transferability of Raman models can be improved using single high-throughput datasets for model calibration through variable-specific preprocessing strategies. Crucially, our approach demonstrates that even widely adopted Orthogonal Partial Least Squares (OPLS) workflows can be rendered robustly transferable, when spectral data are appropriately preprocessed before modeling.

In this study, we assess the transferability of Raman-based models between a high-throughput mini-bioreactor system and a bench-scale bioreactor that fundamentally involves cross-condition challenges. Specifically, the composite challenge of cross-scale transfer is addressed, which in this study inherently includes cross-instrument variability (e.g., different optical probes and detectors). We (i) benchmark the cross-scale performance limitations of conventional single-scale and combined-scale models, and (ii) demonstrate the effectiveness of our proposed methodological approach by identifying and validating variable-specific preprocessing pipelines that strengthen cross-scale robustness using single-scale data for model calibration.

## Methods and materials

### Cell line and cell culture experiments

A stable CHO-DG44 cell line producing an enzyme was cultured in commercial chemically defined basal and feed media. A total of 30 experiments were conducted across two different scales: 23 batches in an Ambr^®^ 250 high-throughput mini-bioreactor system and seven batches in 5 L bench-scale bioreactors. The 23 Ambr^®^ 250 batches served as the primary calibration dataset for Raman model development. Of the seven bench-scale batches, two were used as additional calibration batches for the combined-scale models, whereas the remaining five were used as bench-scale prediction batches for cross-scale performance assessment and preprocessing pipeline comparison. To generate a diverse calibration dataset for Raman modeling, a Design of Experiments (DoE) approach was implemented across the Ambr^®^ 250 batches by varying key process parameters. The complete batch structure and relative operating conditions for both Ambr^®^ 250 and bench-scale experiments are summarized in Suppl. Table 1 using coded parameter levels. Within each process parameter, identical coded levels indicate the same operating condition across batches.

### Cell culture assay and data normalization

Viable cell density (VCD) and cell viability were monitored throughout the cultivation of each batch using a Cedex^®^ HiRes Analyzer (Roche CustomBiotech, Penzberg, Germany). In addition, key metabolites including glucose, lactate, glutamine, glutamate, ammonia, and lactate dehydrogenase were analyzed using a Cedex^®^ Bio HT analyzer (Roche CustomBiotech, Penzberg, Germany). Reference measurements for the Y-block of the Raman calibration models were collected daily from the bioreactors and aligned with spectral acquisitions. For model fitting, each target output was scaled to the [0,1] range using min–max normalization. The minimum and maximum values used for scaling were calculated from the calibration dataset used to fit each model, and the same scaling parameters were then applied to the corresponding prediction dataset.

### Raman spectral data acquisition

Raman spectra were collected at regular intervals throughout the CHO cell culture process, and ambient light interference was minimized during spectral acquisition (Rowland-Jones et al. 2021). Data were acquired using two different Raman spectroscopy systems. For the Ambr^®^ 250 HT system, an at-line BioPAT^®^ spectro (Sartorius, Göttingen, Germany and Endress+Hauser, Reinach, Switzerland) collected spectra via 10 scans with a 30-second exposure time per scan, while for the bench-scale bioreactor, an in-line Raman Rxn2 analyzer (Endress+Hauser, Reinach, Switzerland) acquired spectra via 75 scans with a 10-second exposure time per scan. Although the acquisition parameters differed, both systems operated with a 785 nm laser excitation wavelength, 400 mW laser power, and a spectral coverage of 100–3425 cm⁻¹. For all subsequent model development, the spectral range was limited to 450–1800 cm⁻¹.

### Spectral data preprocessing

Prior to preprocessing, all spectra underwent a standardized quality control to remove spikes and other pronounced artifacts (Suppl. Figure 1). Raw Raman spectra were then preprocessed to correct for artifacts not directly related to chemical composition. Several established techniques were evaluated based on their common application in bioprocess modeling (Liu et al. 2021; Rafferty et al. 2020; Eyster et al. 2021; Webster et al. 2021). Baseline variations were corrected using (1) the first derivative to remove simple additive shifts and drifts, while (2) the Asymmetric Least Squares (ALS) algorithm was employed for more complex, non-linear fluorescence backgrounds. Multiplicative effects arising from light scattering were corrected using (3) the Standard Normal Variate (SNV) transformation. The detailed parameter settings used for each preprocessing method were as follows. The first derivative was calculated using a Savitzky-Golay algorithm with a second-order polynomial and a 15-point window. For ALS baseline correction, the smoothing parameter (λ) was set to 10,000, and the asymmetry parameter (p) was set to 0.001. SNV transformation was applied to each individual spectrum without additional tunable hyperparameters. These preprocessing settings were kept constant across target outputs when a given preprocessing method was included in the corresponding pipeline. The mathematical expressions used for the preprocessing methods are provided below: Eq. (1) describes the first derivative preprocessing, Eq. (2) describes ALS baseline correction, and Eq. (3) describes SNV transformation.

1 $$ y_{i}{'} = \frac{{dy}}{{dx}} \approx \frac{{y_{{i + 1}} - y_{{i - 1}} }}{{2\Delta x}} $$

For the first derivative preprocessing in Eq. (1), where $\:{y}_{i}^{{\prime\:}}$ is the first derivative value at the * i*-th data point, $\:{y}_{i+1}$ and $\:{y}_{i-1}$ are values at adjacent point, and $\:\varDelta\:x$ is the interval between data points.

2 $$ \min \sum\limits_{{i = 1}}^{n} {\left[ {w_{i} \left( {y_{i} - z_{i} } \right)^{2} + \lambda \left( {D^{2} z} \right)_{i}^{2} } \right]} $$

For ALS baseline correction in Eq. (2), where $\:{y}_{i}$ is the original spectral data, $\:{z}_{i}$ is the estimated baseline, $\:{w}_{i}$ is the weight for the residual, $\:\lambda\:$ is the smoothing parameter, $\:{D}^{2}$ is the second-order difference matrix, and * n* is the total number of data points.

3 $$ y_{i}^{'} = \frac{{y_{i} - \bar{y}}}{{\sigma _{y} }} $$

For SNV transformation in Eq. (3), where $\:{y}_{i}^{{\prime\:}}$ is the normalized * i*-th data point, $\:{y}_{i}$ is the original * i*-th data point, $\:\stackrel{-}{y}$ is the mean value of the spectrum, and $\:{\sigma\:}_{y}$ is its standard deviation.

### Chemometric model development and validation

All chemometric modeling, including the preprocessing steps described in “Spectral data preprocessing ” section and the subsequent OPLS regression and validation procedures, was conducted using SIMCA^®^ software (version 17, Sartorius; Göttingen, Germany). The dataset allocation differed according to the modeling strategy. For the standard single-scale models, the 23 Ambr^®^ 250 batches were used for calibration, and five bench-scale batches were used as the cross-scale prediction dataset. For the combined-scale models, calibration was performed using the 23 Ambr^®^ 250 batches together with two bench-scale batches; the remaining five bench-scale batches, which were independent of the bench-scale calibration batches, were used for prediction. For the variable-specific single-scale models, calibration was again performed using only the 23 Ambr^®^ 250 batches, while the five bench-scale batches were used for cross-scale prediction and preprocessing pipeline comparison based on RMSEP and bias. The bench-scale prediction batches were not used for model calibration, latent variable optimization, or coefficient estimation, but were subsequently used to compare preprocessing pipelines based on cross-scale prediction performance. The preprocessing pipeline selection was performed using a constrained systematic screening procedure rather than automated optimization. Candidate pipelines were predefined using combinations of established preprocessing methods described in “Spectral data preprocessing ” section, including the first derivative, SNV, and ALS. For each target output, OPLS models were constructed using the Ambr^®^ 250 calibration dataset and evaluated using a common modeling workflow. The final preprocessing pipeline was selected primarily based on cross-scale prediction performance, particularly RMSEP and bias, using the bench-scale prediction dataset. Spectral diagnostics, including VIP and coefficient-derived Raman region analysis, together with model parsimony, were used as supporting criteria when differences in prediction performance between candidate pipelines were marginal. Using the training set, OPLS regression models were developed to establish a quantitative relationship between the preprocessed Raman spectra (X-block) and the reference measurements (Y-block). The optimal number of latent variables (LVs) for each model was determined using 7-fold cross-validation within the calibration dataset. Cross-validation was used for LV selection and internal calibration-set assessment, whereas cross-scale model transferability was evaluated separately using the bench-scale prediction dataset. To assess whether the 7-fold cross-validation results were affected by potential within-batch correlations in the time-course Ambr^®^ 250 dataset, an additional leave-one-group-out (LOGO) cross-validation analysis was performed as a supplementary sensitivity analysis for four representative Ambr^®^ 250 single-scale calibration models: VCD, glucose, lactate, and glutamate. These target outputs were selected because Raman region information relevant to their spectral interpretation was available from previous studies. In the LOGO analysis, the 23 Ambr^®^ 250 culture batches were used as groups, and all samples from the same batch were kept together during cross-validation.

A clear distinction was maintained between internal validation metrics (R², Q², and RMSEcv) from the calibration dataset and cross-scale prediction metrics (RMSEP and bias) from the bench-scale prediction dataset. The performance was evaluated using key metrics, including (4) the coefficient of determination (R²), (5) the coefficient of cross-validation (Q²), (6) root mean square error (RMSEcv and RMSEP), and (7) bias. Because the target outputs differed substantially in units and observed ranges, RMSECV and RMSEP values are reported as relative percentages. For each target output, the relative RMSE (%) was calculated as the absolute RMSECV or RMSEP divided by the observed range of the corresponding measured reference values and multiplied by 100. The equations for these performance metrics are provided below:

4 $$R^{2} = 1 - \frac{{\sum\limits_{{i = 1}}^{N} {\left( {y_{i} - \hat{{y_{i} }}} \right)^{2} } }}{{\sum\limits_{{i = 1}}^{N} {\left( {y_{i} - \bar{y}} \right)^{2} } }} $$

For coefficient of determination (R²) in Eq. (4), where $\:{y}_{i}$ is the actual value, $\:{\widehat{y}}_{i}\:$is the predicted value from the model, $\:\stackrel{-}{y}$ is the mean of the actual values, and * N* is the total number of data points.

5 $$ Q2 = 1 - \frac{{\sum\limits_{{i = 1}}^{N} {\left( {y_{i} - \hat{y}_{{cv,i}} } \right)^{2} } }}{{\sum\limits_{{i = 1}}^{N} {\left( {y_{i} - \bar{y}} \right)^{2} } }} $$

For coefficient of cross-validation (Q²) in Eq. (5), where $\:{\widehat{y}}_{cv,i}$ is the predicted value for the * i*-th sample when it was held out during the cross-validation process.

6 $$ {{RMSEcv} \mathord{\left/ {\vphantom {{RMSEcv} {RMSEP}}} \right. \kern-\nulldelimiterspace} {RMSEP}} = \sqrt {\frac{1}{N}\sum\limits_{{i = 1}}^{N} {\left( {y_{i} - \hat{y}_{i} } \right)^{2} } } $$

For root mean square error (RMSEcv and RMSEP) in Eq. (6), where $\:{\widehat{y}}_{i}$ is the predicted value obtained during the cross-validation process (in RMSEcv) or from the fitted model applied to the corresponding prediction dataset for RMSEP, and * N* is the total number of data points.

7 $$Bias = \frac{1}{N}\sum\limits_{{i = 1}}^{N} {\left( {y_{i} - ~\hat{{y_{i} }}} \right)} $$

For bias in Eq. (7), where $\:{y}_{i}$ is the actual value, $\:{\widehat{y}}_{i}$ is the predicted value, and * N* is the total number of data points.

### Spectroscopic interpretation of model-relevant Raman regions

To further investigate the spectroscopic basis of preprocessing-dependent model behavior, variable importance in projection (VIP) scores and model coefficient profiles were analyzed for representative target analytes and process attributes. This analysis focused on glucose, lactate, glutamate, and VCD, for which literature-reported Raman shift regions could be reasonably compared with the model-derived spectral regions. VIP scores were first examined across the Raman spectral range of 450–1800 cm⁻¹. Raman shift regions with VIP scores greater than 1 were selected to evaluate whether high-VIP regions could distinguish target-specific Raman contributions. For Suppl. Figure 2, model coefficient values were then visualized within these VIP > 1 regions to compare coefficient patterns across representative target outputs.

**Table 1:** Comparison of top-coefficient-derived Raman shift regions with literature-reported Raman regions for representative target outputs

Target Output	Coefficient-derived Raman regions (cm^− 1^)		Literature-reported Raman regions (cm^− 1^)	References
	Std. preprocessing	Opt. preprocessing		
Glucose	525–530	526–529	510–518	(Goldrick et al. 2020; Mathlouthi and Luu 1980; Singh et al. 2015; Vasko et al. 1971)
	891–894	891–894	893–914	(Barrett 1981; De Gelder et al. 2007; Goldrick et al. 2020; Mathlouthi and Luu 1980)
	–	1104–1105	–	–
	1133–1142	1134–1141	1120–1130	(Barrett 1981; De Gelder et al. 2007; Goldrick et al. 2020; Kačuráková and Mathlouthi 1996; Mathlouthi and Luu 1980; Singh et al. 2015; Vasko et al. 1971)
Lactate	846–855	845–855	855–860	(Cassanas et al. 1991; Goldrick et al. 2020; Pecul et al. 2002; Singh et al. 2015)
	861–870	861–869		
Glutamate	866–868	–	–	–
	1010–1013	1009–1012	–	–
	–	1394–1406	1401–1434	(De Gelder et al. 2007; Shurvell and Bergin 1989)
	1421	–		
	1431–1435	1432–1434		
VCD	525–529	–	–	–
	894	892–896	–	–
	–	–	1003 [phenylalanine]	(Raj et al. 2025; Sofińska et al. 2024)
	–	1082–1085	–	–
	1103–1108	1105–1107	1076, 1095 [Nucleic acid]	(Dong et al. 2024; Notingher 2007)
	1134–1141	1136–1139	–	–

Raman shift regions were evaluated for glucose, lactate, glutamate, and VCD, for which literature-reported Raman regions could be reasonably compared with the model-derived spectral regions. For each model, coefficient-derived regions were identified by selecting the regions containing the 20 highest absolute coefficient values. Regions obtained using the standard preprocessing pipeline and the optimized variable-specific preprocessing pipeline are shown and compared with Raman regions previously reported in the literature. For VCD, the literature comparison was based on biomass-associated spectral regions rather than unique VCD-specific molecular peaks, because VCD reflects aggregate cellular biomass rather than a single molecular species

For the coefficient-based literature comparison summarized in Table 1, Raman shift regions were identified separately by selecting the regions containing the 20 highest absolute coefficient values from each model. These top-coefficient-derived regions were compared between the standard and optimized preprocessing pipelines and further compared with Raman shift regions previously reported in the literature for the corresponding target outputs. For VCD, the literature comparison was based on biomass-associated spectral regions rather than unique molecular Raman peaks, because VCD reflects aggregate cellular biomass rather than a single molecular species (Dong et al. 2024).

**Table 2:** Preprocessing pipeline screening candidates and selection for optimal variable-specific Raman models

Target output	RMSEP, %				Final selected pipeline	$\:\varDelta\:$RMSEP (%)
	Std. pipeline	Screening pipeline candidates				
	1 st D → S	1 st D → S → A	A → 1 st D → S	S → 1 st D → A		
VCD	6.6	12.9	7.4	22.2	1 st D → S	N/A
Viability	15.8	10.0	11.6	17.5	1 st D → S → A	36.7
Glucose	5.3	7.4	6.0	29.3	1 st D → S	N/A
Lactate	6.4	9.5	6.8	5.8	1 st D → S	N/A
Glutamine	18.0	11.7	13.3	16.5	1 st D → S → A	35.0
Glutamate	13.5	10.6	7.3	50.7	A → 1 st D → S	45.9
Ammonia	10.7	9.2	15.3	53.8	1 st D → S → A	14.0
LDH	10.7	6.0	4.7	19.4	A → 1 st D → S	56.1

This table outlines the various data preprocessing pipelines evaluated for eight target outputs, including process attributes (VCD and viability) and target analytes (glucose, lactate, glutamine, glutamate, ammonia, and LDH). The pipelines combined techniques including the first derivative (1st D), standard normal variate (S), and asymmetric least squares (A). For most target outputs, the final preprocessing pipeline was selected based on its ability to minimize the root mean square error of prediction (RMSEP, %) across scales. For target outputs with robust analyte-associated or biomass-associated spectral responses, the standard pipeline (1st D → S) was optimal, whereas complex target outputs required additional baseline correction. For lactate, the standard pipeline was retained despite a marginally lower RMSEP obtained with S → 1 st D → A, because the additional ALS step did not provide a clear spectroscopic benefit based on coefficient-derived Raman region analysis and increased preprocessing complexity

## Results and discussion

### Single-scale model performance and the cross-scale compatibility challenge

The primary objective of this study was to develop a cross-scale compatible Raman model calibrated using data from the Ambr^®^ 250 high-throughput system and evaluated for prediction performance across scales. As a baseline, a 1 st derivative (Der.) → SNV preprocessing pipeline was adopted as the standard approach (Otto 2023; Singh 2025). Following this preprocessing strategy and the OPLS modeling methodology, individual standard single-scale models were developed for eight target outputs using the 23 Ambr^®^ 250 batches as the calibration dataset.

**Fig. 1:**
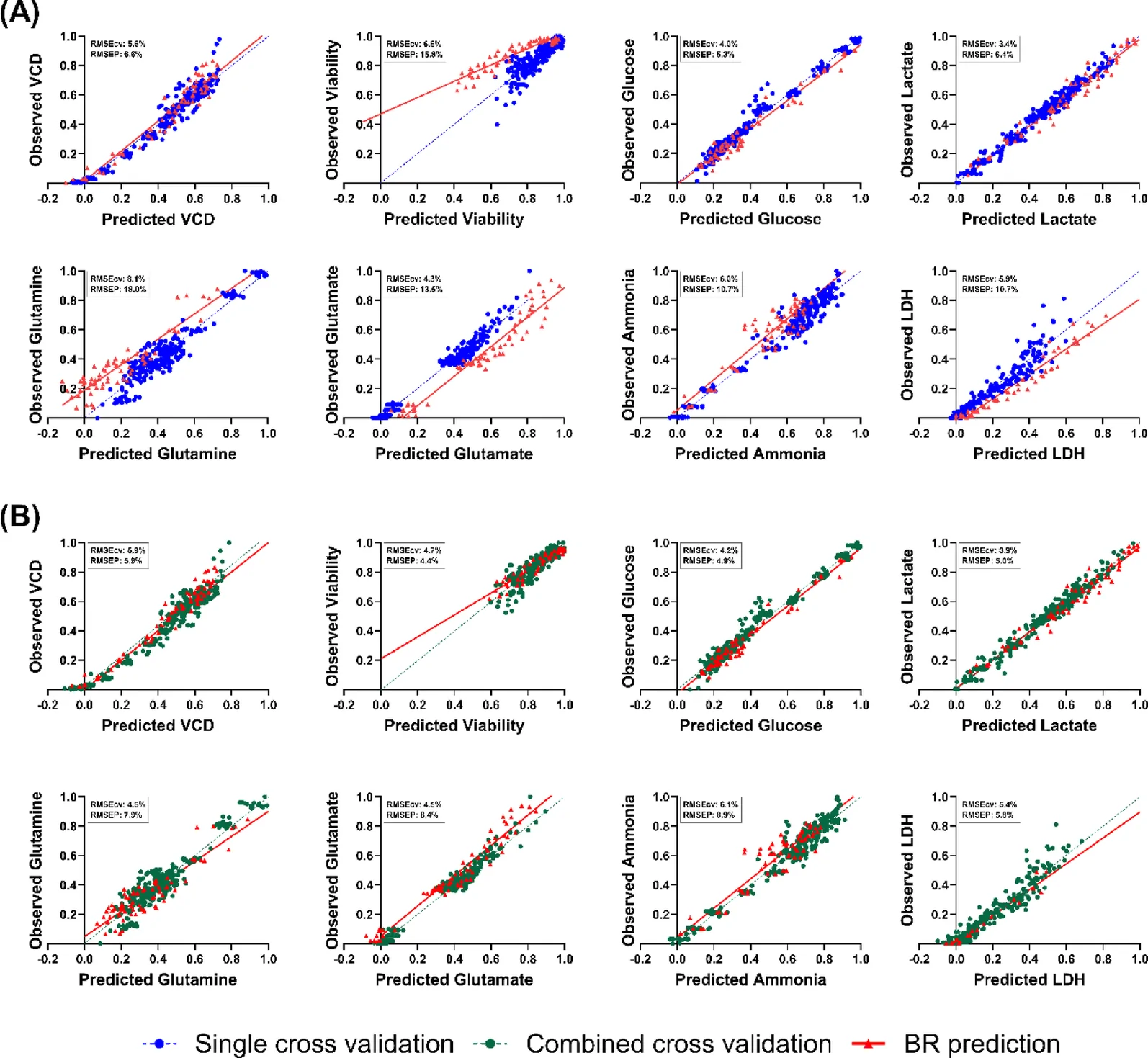
Performance comparison between standard single-scale and combined-scale models for cross-scale monitoring. Cross-validation and cross-scale prediction performance of Raman models. **A** The single-scale model demonstrates internal cross-validation (blue circles) but exhibits poor cross-scale prediction when applied to bench-scale cultures (red triangles). **B** The combined-scale model displays cross-validation (green circles) and significantly improved cross-scale prediction performance for bench-scale cultures (red triangles) compared to the single-scale approach

**Fig. 2:**
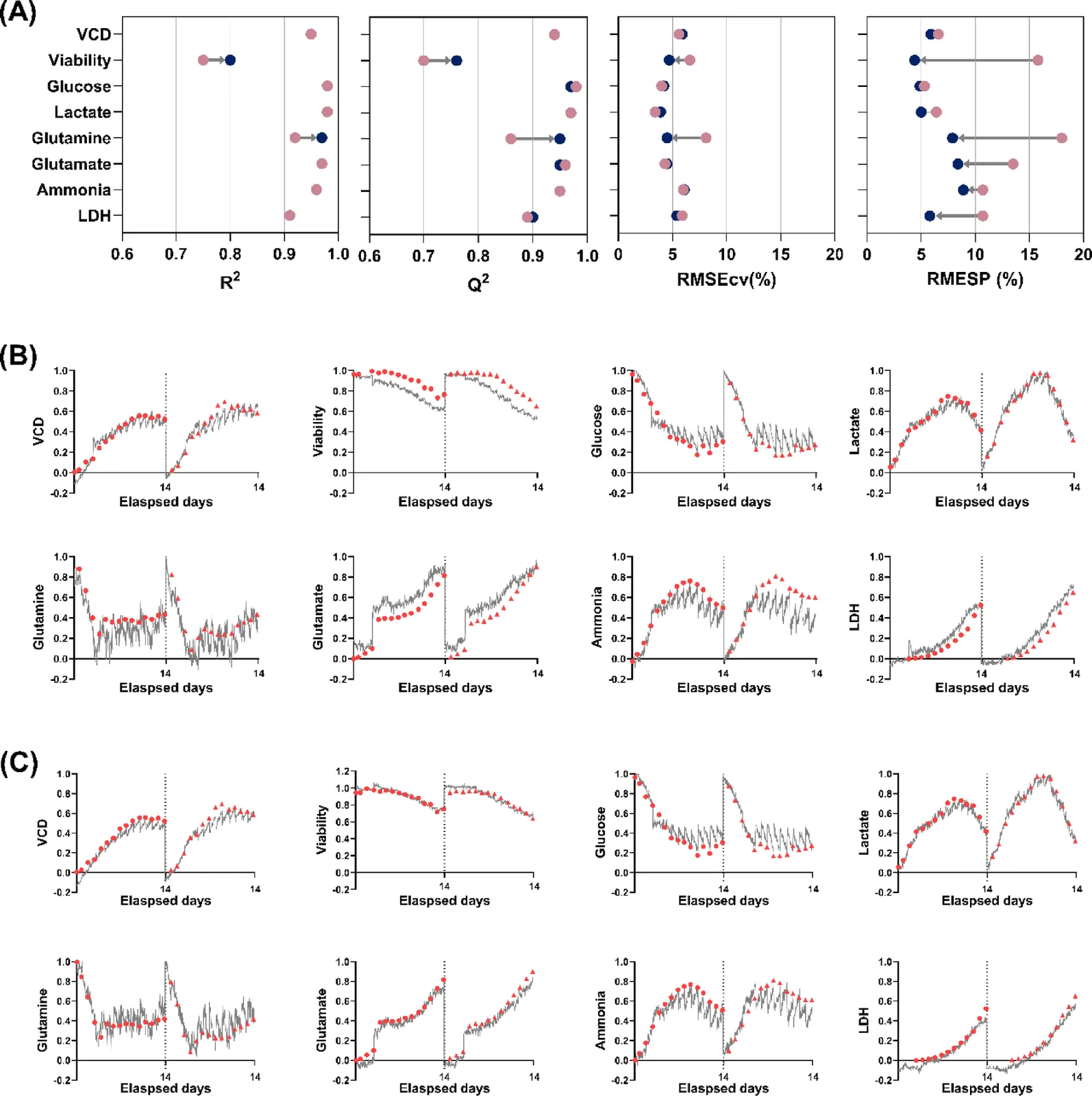
Predictive performance metrics and real-time monitoring evaluation of single-scale and combined-scale models. Quantitative assessment of model transferability to bench- scale systems. **A** Evaluation metrics comparing the single-scale model (pink circles) and the combined-scale model (navy circles). Gray arrows highlight the substantial improvement in RMSEP (%) achieved by the combined-scale model. **B** Real-time monitoring profiles obtained by applying the standard single-scale Ambr^®^ 250-calibrated models to two additional bench-scale bioreactor batches used for real-time monitoring demonstration (*n* = 2 batches). **C** Real-time monitoring profiles obtained by applying the combined-scale models to the same two additional bench-scale bioreactor batches (*n* = 2 batches). The *n* = 2 batches shown in panels **B** and **C** were used to illustrate real-time monitoring behavior and were separate from the five bench-scale prediction batches used for quantitative RMSEP and bias evaluation

The resulting models demonstrated strong internal cross-validation performance within the Ambr^®^ 250 calibration dataset (Fig. 1A). The cross-scale prediction performance was then evaluated by applying the Ambr^®^ 250-trained models to the five bench-scale prediction batches. As summarized in Fig. 2A and Suppl. Table 2, all standard single-scale models exhibited low RMSEcv (%) values (3.4–8.1%), and most achieved R² > 0.90 and Q² > 0.85. The viability model showed relatively lower R² and Q² values (~ 0.70), likely because the viability reference measurements were concentrated within a relatively narrow high-viability range, with fewer samples representing low-viability conditions. In addition, viability is a process attribute rather than a single molecular species with a distinct Raman band; therefore, its prediction is expected to rely mainly on indirect cell-state-associated spectral variation rather than a unique viability-specific Raman signal. These factors can reduce apparent R² and Q² values despite a low RMSECV (%). For four representative Ambr^®^ 250 single-scale calibration models, namely VCD, glucose, lactate, and glutamate, additional LOGO cross-validation using the 23 Ambr^®^ 250 culture batches as groups produced Q² and RMSEcv values highly comparable to those obtained using 7-fold cross-validation. This supplementary analysis indicates that, for these representative target outputs, the internal validation performance was not substantially inflated by within-batch correlations in the Ambr^®^ 250 time-course dataset (Suppl. Table 3). However, despite their strong internal calibration-set performance, several standard single-scale models exhibited markedly reduced predictive performance when applied to the five bench-scale prediction batches (Fig. 1A). This deterioration was accompanied by substantially increased RMSEP (%) values and pronounced systematic biases (Fig. 2A and Suppl. Table 2). Such scale-transfer discrepancies are commonly observed in chemometrics when models encounter out-of-distribution data (Guo et al. 2021). When the standard preprocessing-based single-scale Ambr^®^ models were applied to the bench-scale bioreactor dataset, predictions for VCD, glucose, and lactate showed relatively good agreement with the corresponding bench-scale reference trends. In contrast, predictions for viability, glutamine, glutamate, ammonia, and LDH showed larger discrepancies from the corresponding bench-scale reference measurements (Fig. 2B). This result highlights a key limitation of applying a fixed preprocessing pipeline to Raman datasets obtained under different experimental conditions. The prediction discrepancies can arise from cross-condition spectral variability associated with both volumetric scale differences and differences in Raman instrument optical configurations. Consequently, the standard preprocessing pipeline was insufficient to compensate for these cross-scale and instrument-dependent spectral differences.

### Performance benchmark establishment and key insights into preprocessing strategy

To overcome the limitations of the standard single-scale model and to establish a performance benchmark, a combined-scale modeling approach was employed (Berry et al. 2015; Gibbons et al. 2022). The combined-scale model was calibrated using the 23 Ambr^®^ 250 batches together with two bench-scale calibration batches and was evaluated using five independent bench-scale prediction batches. These two bench-scale calibration batches were not included in the bench-scale prediction dataset. Compared with the standard single-scale Ambr^®^-based model, the combined-scale model demonstrated substantially improved predictive performance for the five bench-scale prediction batches (Fig. 1B). For poorly transferred target outputs such as viability and glutamine, RMSEP (%) improved by factors of 1.2–3.5 (Fig. 2A), while the bias for glutamate, ammonia, and LDH was reduced by approximately 30% on average (Suppl. Table 2). Furthermore, evaluation of the prediction profiles for the five bench-scale prediction batches confirmed that the combined-scale model improved predictive performance for all target outputs that previously exhibited reduced prediction accuracy in the standard single-scale model (Fig. 2C).

The superior performance of the combined-scale model provides insight into the underlying cause of the standard single-scale model’s failure. Based on the observations described in “Single-scale model performance and the cross-scale compatibility Challenge” section, we hypothesized that the use of a universal preprocessing pipeline may be particularly disadvantageous for target outputs characterized by weak or highly overlapping spectral signals. Unlike glucose and lactate, which are major metabolites with relatively robust Raman responses, and VCD, which reflects aggregate biomass-associated spectral variation, poorly transferred target outputs (viability, glutamine, glutamate, ammonia, and LDH) are present at lower concentrations, show limited dynamic variation, or exhibit more indirect and overlapping spectral responses. Moreover, their spectral signatures likely overlap extensively with those of background components, including complex media constituents and host cell proteins (HCPs). As a result, the analytical signals associated with these target outputs may be partially obscured by background spectral features arising from the complex cell culture matrix, including overlapping Raman bands and fluorescence-related contributions.

Building on these observations, we formulated a mechanistic hypothesis to explain the failure of the standard single-scale model. Because the model was trained exclusively on Ambr^®^ 250 data, it may have captured not only chemically meaningful spectral information but also scale-dependent spectral patterns, such as fluorescence baselines or impurity-related signals, that coincidentally correlated with the reference measurements within that dataset. Such correlations are likely dataset-specific rather than mechanistically meaningful and therefore may not transfer reliably to bench-scale conditions.

Within this framework, the improved performance of the combined-scale model can be interpreted as a consequence of disrupting these scale-dependent correlations. By integrating spectrally distinct datasets obtained from two different process scales (Suppl. Figure 3), the spectral variance of the calibration set was substantially expanded. This broader variance structure reduces the influence of spectral features that are not consistently associated with the reference measurements across scales while favoring latent structures that remain predictive in both datasets. Consequently, the model becomes more strongly driven by chemically meaningful information shared between scales rather than by scale-dependent artifacts.

This hypothesis can be further examined by testing the inverse scenario. If spectral variance is artificially compressed, the resulting dataset should reproduce the type of information distortion observed in the single-scale model and consequently deteriorate model performance. To evaluate this effect, the order of preprocessing operations was intentionally altered by applying SNV prior to the 1 st derivative. Because SNV normalizes each spectrum by its own standard deviation, both chemical signals and spectral artifacts are subjected to the same normalization process. When weak chemical signals coexist with large fluorescence baselines, the calculated standard deviation can become disproportionately influenced by these baseline contributions. Under such conditions, SNV may effectively over-normalize the spectra, compressing the overall signal space.

**Fig. 3:**
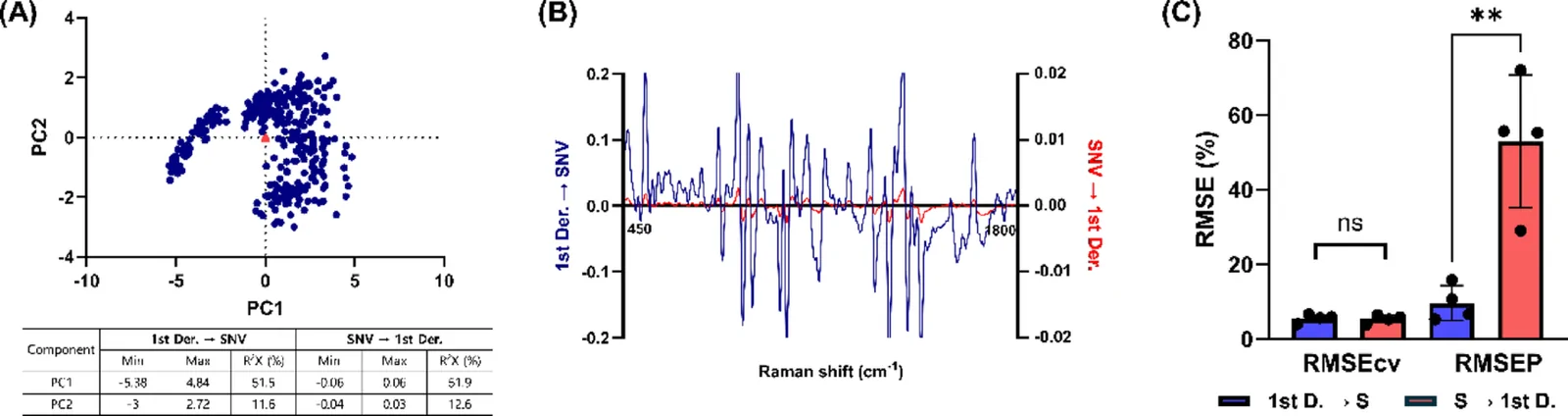
Effect of altered preprocessing order on spectral variance and cross-scale model transferability. Impact of standard normal variate (SNV) application timing. **A** PCA score plots of the calibration dataset comparing the standard 1 st Der. → SNV pipeline (blue circles) against the SNV → 1 st Der. pipeline (red triangles). The reverse pipeline artificially clusters the data structure around the origin. **B** OPLS Y-related predictive profiles for independent bench-scale data. The model using the standard pipeline (left Y-axis, blue line) maintains a full dynamic range. Conversely, the reverse pipeline (right Y-axis, red line) exhibits a severely compressed dynamic range, indicating a distortion of predictive information. Apparent visual data harmonization in PCA does not ensure predictive robustness. **C** Cross-scale prediction performance comparison showing that altering the preprocessing order does not impact internal validation but drastically increases cross-scale prediction errors (standard preprocessing: blue bar; reverse preprocessing: red bar)

This signal compression is reflected in the PCA score plot (Fig. 3A), which appears visually well aligned, and in the OPLS predictive profile (Fig. 3B), where the dynamic range of the predictive signal is markedly distorted relative to that obtained with the standard preprocessing pipeline. Importantly, the narrowed variance observed here does not necessarily indicate a complete loss of information. Rather, it illustrates how universal preprocessing can disproportionately compress or distort variance associated with specific analytes relative to their original spectral signatures. Such structural distortion ultimately compromises the model’s ability to transfer across different process scales.

Although the compressed model still shows seemingly acceptable calibration performance (low RMSEcv (%)), its reliance on distorted variance structures leads to a complete loss of predictive capability when applied to an out-of-distribution dataset, namely the bench-scale data. Even the VCD and glucose models, which exhibited low RMSEP (%) under the standard preprocessing pipeline, showed substantial performance degradation under this modified preprocessing order (Fig. 3C). This contrast highlights that satisfactory calibration statistics alone do not guarantee robust model transferability.

Taken together, these findings suggest that the transferability of spectroscopic models is strongly influenced by how preprocessing operations shape the variance structure of the calibration dataset. Preserving predictive signal dynamics appears to be more critical for cross-scale robustness than achieving simple visual alignment of spectra.

### Cross-scale prediction improvement by variable-specific preprocessing

The insights derived from “Performance benchmark establishment and key insights into preprocessing strategy” section highlight an inherent limitation of the combined-scale modeling approach. In the combined-scale model, spectral variance is expanded by integrating spectra obtained from different process scales, thereby exposing the model to a broader range of spectral variations and improving its robustness. Although this variance-expansion strategy proved effective, it is often impractical in real process development environments where multi-scale experimental data are limited. Therefore, the primary objective of this study was to determine whether comparable cross-scale robustness could be achieved using single-scale data for model calibration.

Rather than expanding spectral variance by incorporating spectra from additional process scales, the strategy proposed here focuses on reducing the influence of confounding background spectral variation while preserving chemically meaningful spectral information. In particular, background fluorescence and overlapping spectral contributions associated with host cell proteins (HCPs) and complex media components represent dominant sources of spectral interference in cell culture fluid (CCF) spectra. These background contributions can obscure weak Raman signals associated with several processattributes and thereby hinder reliable model transferability.

**Fig. 4:**
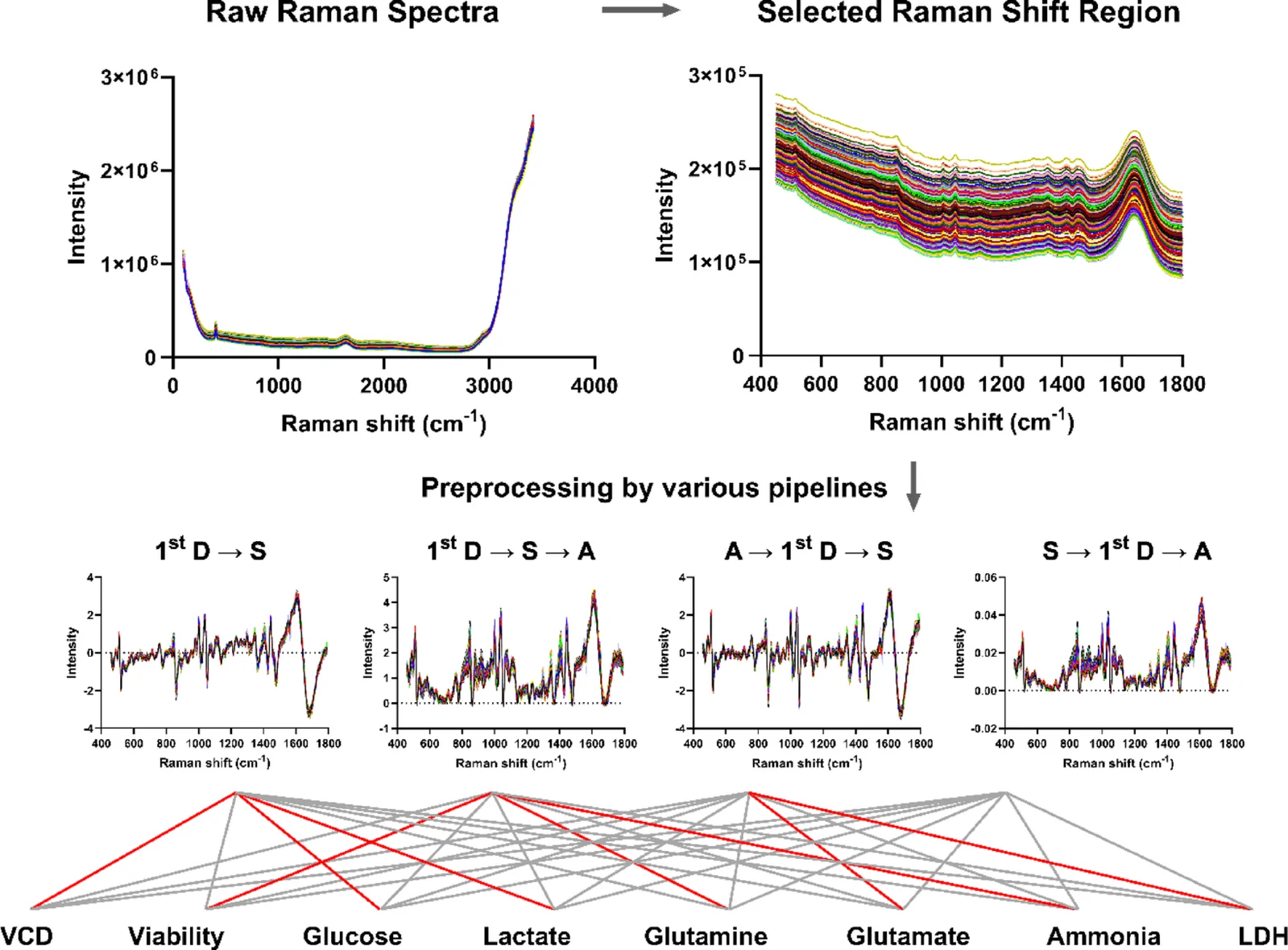
Schematic workflow of variable-specific preprocessing pipelines for single-scale Raman model optimization. Systematic screening flow for variable-specific preprocessing. First, a specific spectral region (450 to 1800 cm^− 1^) is selected from the full raw Raman spectra. Next, four distinct preprocessing pipelines are applied to the spectral data. Finally, RMSEP and spectroscopic interpretability were evaluated to select the final preprocessing pipeline (red line) for each target output

**Fig. 5:**
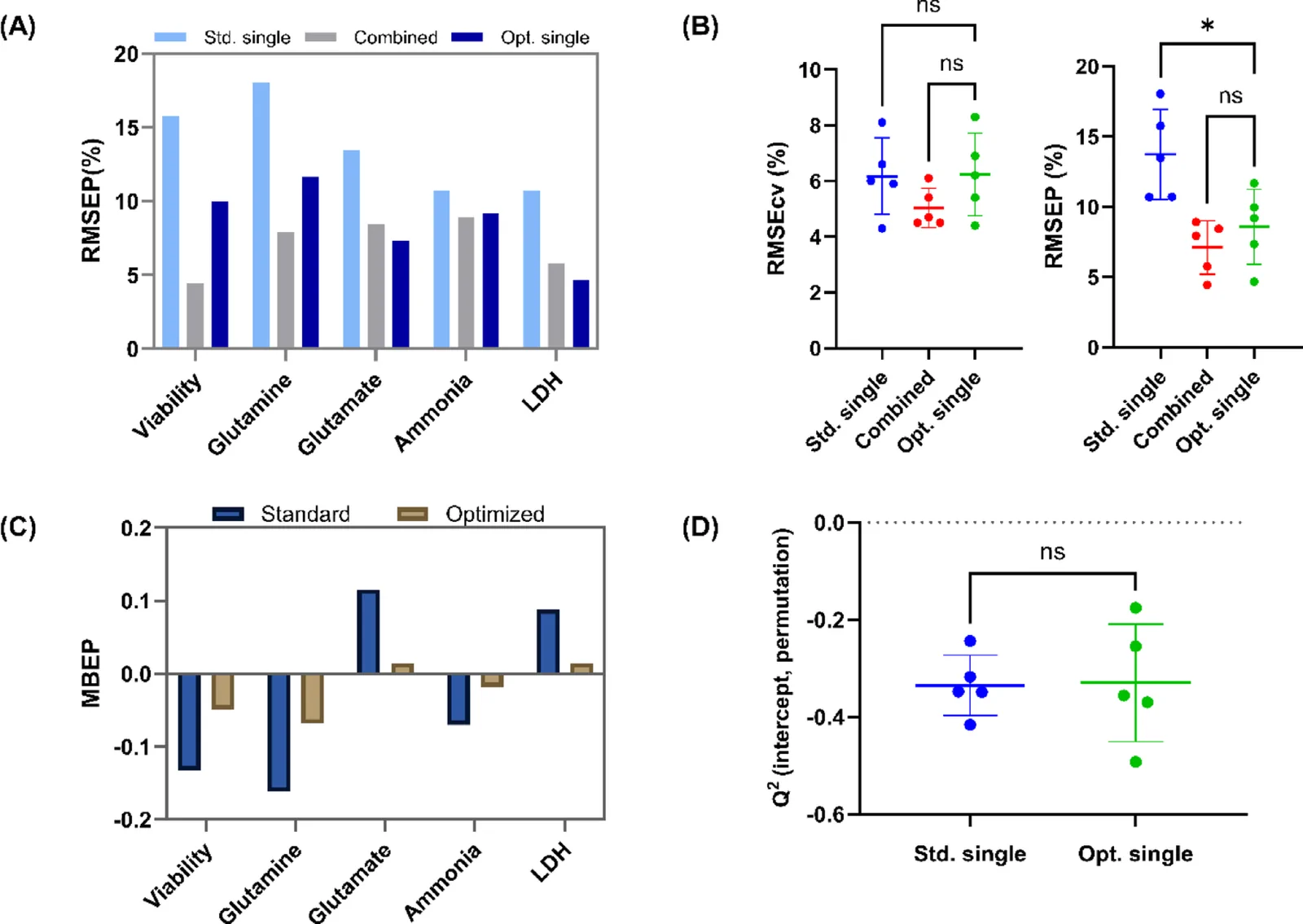
Cross-scale predictive performance of single-scale models using optimized variable-specific preprocessing pipelines. Comparison of predictive capabilities among standard single-scale, combined-scale, and optimized single-scale models. **A** Cross-scale prediction error (RMSEP, %) of the standard single-scale (sky blue), combined-scale (gray), and optimized single-scale (blue) models. **B** Paired t-test results for internal (RMSEcv) and cross-scale (RMSEP) errors across eight target outputs. All three models show no statistical difference in internal errors, whereas the standard single-scale model exhibits statistically higher cross-scale prediction errors than the other two. **C** Prediction bias for five target outputs with weak signal characteristics. The optimized single-scale model effectively reduces bias across all parameters compared to the standard model. **D** Permutation tests (100 permutations) for the five weak-signal target outputs (viability, glutamine, glutamate, ammonia, LDH) using the standard (blue dots) and optimized (green dots) single-scale models. All models yield a negative Q2 from randomly permuted Y-data, confirming statistical validity without chance correlation

To evaluate this hypothesis, multiple preprocessing pipeline combinations were systematically assessed for all target outputs (Fig. 4). For each candidate pipeline, the variable-specific single-scale models were calibrated using only the 23 Ambr^®^ 250 batches, and the five bench-scale prediction batches were used to compare cross-scale prediction performance based on RMSEP and bias. These bench-scale prediction batches were not used for coefficient estimation or LV selection. The final preprocessing pipeline for each target output was selected primarily according to cross-scale RMSEP and bias, with coefficient-derived spectral interpretability (Suppl. Figure 2) and model parsimony used as secondary criteria. Thus, the optimized preprocessing pipelines should be interpreted as empirically selected cross-scale prediction strategies rather than outputs of an automated preprocessing optimization algorithm. The cross-scale predictive performance of each optimized single-scale model, expressed as RMSEP (%), was compared with that of the standard single-scale model and the combined-scale benchmark model using the same five bench-scale prediction batches (Fig. 5).

Across the five initially poorly transferable target outputs, the optimized variable-specific preprocessing pipelines reduced RMSEP (%) from 15.8 to 10.0 for viability, 18.0 to 11.7 for glutamine, 13.5 to 7.3 for glutamate, 10.7 to 9.2 for ammonia, and 10.7 to 4.7 for LDH. These changes correspond to RMSEP reductions of 36.7%, 35.0%, 45.9%, 14.0%, and 56.1%, respectively, relative to the standard preprocessing pipeline (Table 2). The quantitative RMSEP reduction relative to the standard single-scale model and the degree of performance gap closure relative to the combined-scale benchmark are summarized in Suppl. Table 4.

The results showed that the optimal preprocessing strategy depended strongly on the spectral characteristics of each target output. For glucose and lactate, which exhibit relatively robust metabolite-associated Raman responses, and for VCD, which reflects aggregate biomass-associated spectral variation, the standard preprocessing pipeline (1st Der. → SNV) already provided robust predictive performance. Additional preprocessing steps did not improve prediction accuracy and in some cases even degraded model performance. While the SNV → 1 st Der. → ALS pipeline yielded a slightly lower RMSEP for lactate than the standard pipeline (5.8% versus 6.4%), the coefficient-derived Raman regions were largely similar between the two approaches (Table 1). Therefore, the standard pipeline was retained for lactate, as the marginal improvement in RMSEP did not justify the additional preprocessing step, considering model parsimony and spectral interpretability. This result suggests that, when analyte-associated Raman responses are relatively robust, as observed for glucose and lactate, or when prediction is driven by broad aggregate biomass-associated spectral variation, as observed for VCD, the standard 1 st Der. → SNV pipeline can sufficiently preserve predictive spectral contrast while correcting major baseline and scattering-related effects. In these cases, additional asymmetric least squares (ALS) correction may not improve cross-scale prediction because part of the broad predictive variation may be modeled as baseline rather than retained as Y-correlated spectral information.

In contrast, five target outputs (viability, glutamine, glutamate, ammonia, and LDH) exhibited relatively poor cross-scale predictive performance despite acceptable internal validation results when the standard preprocessing pipeline was applied. These target outputs are characterized by weak or highly overlapping Raman signals, where complex fluorescence baselines represent a dominant source of spectral interference. When ALS baseline correction was incorporated into the preprocessing pipeline, RMSEP (%) for these target outputs improved substantially (Fig. 5A and B).

### Mechanistic and spectroscopic interpretation of preprocessing effects

The mechanistic basis of the ALS-dependent improvement can be interpreted through its ability to model broad, nonlinear fluorescence-dominated baseline components and reduce their influence on the covariance structure between Raman spectra and reference values. This effect is particularly relevant for weak, indirect, or highly overlapping spectral responses, where analyte-associated variance has a lower effective signal-to-background ratio and can be partially masked by fluorescence- or matrix-related spectral variation.

The impact of ALS extends beyond simple baseline correction. Model bias frequently arises when spurious correlations are formed between systematic background variations, such as fluorescence intensity, and analyte concentrations within the calibration dataset. Such spurious correlations may be amplified in batch cultures because analyte concentrations, cell growth, media consumption, and background spectral features often change concurrently over cultivation time. As illustrated in Fig. 5C, the introduction of ALS substantially reduced model bias. By suppressing fluctuating background signals, ALS limits the formation of such non-causal correlations and stabilizes the covariance structure between the Raman spectral data matrix (X-block) and the reference measurements (Y-block).

From a chemometric perspective, this effect can be interpreted as a restructuring of the X-variance space prior to PLS modeling. By reducing variance components associated with fluorescence background and other non-informative spectral contributions, ALS effectively increases the relative contribution of chemically relevant spectral variance. Consequently, the latent variables extracted by the PLS algorithm are more likely to capture analyte-related spectral information rather than scale-dependent spectral artifacts (Gautam et al. 2015; Rinnan et al. 2009).

It should be emphasized that ALS itself is not a newly proposed preprocessing method but a well-established technique widely used in chemometrics-based Raman analysis. However, the results of this study demonstrate that the effectiveness of such preprocessing approaches strongly depends on the spectral characteristics of the target output. For glucose and lactate, which occur as major metabolites in cell culture fluid and exhibit relatively robust Raman responses, the application of ALS provided little additional benefit (Table 2). VCD was adequately predicted using the standard preprocessing pipeline, although its prediction should be interpreted as arising from aggregate biomass-associated spectral variation rather than a unique VCD-specific molecular Raman band. In contrast, target outputs characterized by weak or overlapping spectral signatures showed clear improvements when ALS was incorporated into the preprocessing pipeline. These observations highlight that preprocessing strategies should not be applied uniformly across all target outputs but rather tailored according to their spectral characteristics. In this context, ALS represents one example of a variable-specific preprocessing strategy capable of improving cross-scale model robustness. To further examine whether the preprocessing-dependent improvements were supported by spectroscopically meaningful information, VIP- and coefficient-based analyses were performed for representative target analytes with literature-comparable Raman regions, namely glucose, lactate, glutamate, and VCD. The Raman shift regions with VIP scores greater than 1 were broadly shared across these target analytes (Suppl. Figure 2A). This result suggests that VIP scores mainly reflected common process-related spectral variance present during cell culture progression, rather than clearly resolving target-specific Raman regions. This behavior is reasonable in batch cell culture datasets, where several metabolites and cellular attributes change concurrently over cultivation time.

Therefore, model coefficient values were additionally examined within the VIP > 1 Raman shift regions to visualize whether coefficient patterns showed more target-dependent behavior than VIP scores alone (Suppl. Figure 2B). In contrast to the VIP-based pattern, the coefficient heatmap revealed more target-dependent spectral contributions. For direct comparison with literature-reported Raman regions, coefficient-derived Raman regions were identified separately by selecting the regions containing the 20 highest absolute coefficient values from each model. As summarized in Table 1, glucose and lactate showed top-coefficient-derived Raman regions that overlapped with literature-reported regions (Barrett 1981; Cassanas et al. 1991; De Gelder et al. 2007; Goldrick et al. 2020; Kačuráková and Mathlouthi 1996; Mathlouthi and Luu 1980; Pecul et al. 2002; Singh et al. 2015; Vasko et al. 1971) even under the standard preprocessing pipeline, which is consistent with their relatively strong and well-resolved Raman signals. In contrast, glutamate showed broadly distributed top-coefficient-derived regions under the standard preprocessing pipeline, whereas the optimized pipeline produced a more focused set of Raman regions that overlapped with literature-reported glutamate-associated bands (De Gelder et al. 2007; Shurvell and Bergin 1989). For VCD, the standard preprocessing pipeline emphasized regions similar to those observed for glucose, whereas the optimized pipeline highlighted regions more consistent with biomass-associated spectral features reported in previous Raman studies (Dong et al. 2024; Raj et al. 2025; Sofińska et al. 2024). These VCD-associated regions should be interpreted as aggregate spectral contributions from cellular biomass rather than unique molecular peaks of VCD itself.

These observations suggest that variable-specific preprocessing did not merely reduce prediction error empirically but also altered the spectral contribution structure of the models. In particular, ALS-containing pipelines appeared to suppress broadband baseline-dominated contributions and enhance the relative contribution of chemically meaningful Raman regions. Although this analysis does not definitively exclude all time-correlated effects inherent to batch culture datasets, the observed shift in coefficient-derived regions toward literature-reported analyte-associated bands provides additional evidence that the optimized preprocessing pipelines reduced the influence of non-specific process-related variation and improved the extraction of spectroscopically meaningful information.

These findings were further supported by OPLS-based diagnostic analysis (Table 3). The five bench-scale prediction batches were applied to the fitted standard single-scale and optimized single-scale OPLS models to examine the relationship between cross-scale prediction performance and spectral variance diagnostics. The optimized models consistently showed substantial improvements in predictive accuracy (RMSEP, Fig. 5B) and bias (Fig. 5C) compared with the standard model. However, the predictive-to-orthogonal X-variance ratio, calculated as R²X[1]/R²Xo[1], exhibited target-dependent behavior. As shown in Table 3, this ratio decreased for viability and glutamate, increased for glutamine, and remained largely unchanged for ammonia and LDH. These results suggest that X-variance-based diagnostic indicators alone may not provide a consistent interpretation when evaluating cross-scale model transferability. Because models handle scale-dependent spectral artifacts differently depending on the target output, this complexity cannot be fully captured by simple X-variance metrics. Therefore, Y-based cross-scale prediction metrics, particularly RMSEP and bias, provide a more direct and reliable basis for evaluating cross-scale model robustness.

**Table 3:** Orthogonal partial least squares (OPLS) diagnostic analysis of X-variance partitioning

Target output	Variance explained by predictive comp. (*R*^2^X[1])		Variance explained by orthogonal comp. (*R*^2^Xo[1])		*R*²X[1]/*R*²Xo[1]	
	Std. pipeline	Opt. pipeline	Std. pipeline	Opt. pipeline	Std. pipeline	Opt. pipeline
Viability	0.341	0.307	0.225	0.357	1.34	0.86
Glutamine	0.242	0.238	0.279	0.234	0.87	1.02
Glutamate	0.498	0.478	0.113	0.117	4.41	4.09
Ammonia	0.434	0.450	0.142	0.149	3.06	3.02
LDH	0.293	0.290	0.191	0.187	1.53	1.55

OPLS-based diagnostic analysis comparing the standard preprocessing pipeline with the optimized variable-specific pipelines. The table presents the variance explained by the predictive component, the variance explained by the orthogonal component, and the resulting predictive-to-orthogonal X-variance ratio, calculated as R²X[1]/R²Xo[1]. This ratio is used as an OPLS diagnostic indicator and should not be interpreted as an experimental Raman signal-to-noise ratio. Its target-dependent behavior highlights that prediction metrics, such as RMSEP and bias, are more reliable indicators of cross-scale robustness than X-variance-based diagnostics alone

To further verify the statistical validity of the models, permutation tests (*n* = 100) were performed. The original Q² values (Fig. 2A) calculated from the true datasets were significantly positive, whereas models constructed using randomly permuted Y-data consistently produced negative Q² values (Fig. 5D). These results confirm that the predictive performance of both the standard and optimized models was statistically valid and not the result of chance fitting. Although the cross-scale prediction dataset was limited to five bench-scale batches, the statistical significance observed in the paired t-tests (Fig. 5B) and permutation tests indicate that the observed improvements were not attributable to chance fitting alone.

Overall, single-scale models constructed using optimized variable-specific preprocessing pipelines achieved substantially improved predictive accuracy compared with models built using the standard preprocessing strategy and, in several cases, approached the performance of the resource-intensive combined-scale benchmark model. These findings demonstrate that selecting preprocessing strategies according to the spectral characteristics of individual target outputs is a critical determinant of cross-scale Raman model transferability. From a practical perspective, the present results indicate that robust cross-scale Raman models can be developed using high-throughput mini-bioreactor data when appropriate variable-specific preprocessing strategies are applied. Such an approach may substantially reduce the reliance on large-scale experiments during PAT model development and provide a cost-efficient and agile modeling strategy for modern biopharmaceutical process development.

## Conclusion

This study addresses the critical challenge of cross-scale compatibility in Raman-based monitoring of CHO cell culture processes. Standard single-scale models showed limited transferability between process scales, whereas combined-scale models improved prediction performance but required additional multi-scale experimental data, limiting their practicality during early-stage development.

By strategically optimizing the spectral preprocessing pipeline, we demonstrate that models calibrated using mini-scale data can achieve reliable cross-scale prediction performance, in some cases approaching that of combined-scale benchmark models. Importantly, the optimal preprocessing strategy was target output-dependent and determined by the interaction between Raman signal characteristics and dominant spectral interferences in the cell culture matrix. Rather than applying a universal preprocessing pipeline, selecting preprocessing methods that preserve analyte-related spectral variance proved critical for improving model transferability.

From a practical perspective, this approach enables the development of robust and transferable Raman-based PAT models using high-throughput mini-bioreactor systems, thereby reducing the reliance on resource-intensive large-scale experiments during process development. Within the broader analytical framework for Raman-based bioprocess monitoring (Guo et al. 2021), this strategy provides a rapid and experimentally efficient alternative to approaches based on culture-free chemical mixtures (Hara et al. 2023). By enabling earlier and more reliable implementation of spectroscopic monitoring, the methodology supports Quality-by-Design (QbD) principles and facilitates more agile biomanufacturing processes.

Although the workflow was demonstrated using a CHO-DG44 fed-batch process and Raman spectroscopy, its applicability to other mammalian cell lines, media formulations, culture modes such as perfusion, and other spectroscopic platforms requires further validation. In this context, future work will focus on integrating the optimized variable-specific preprocessing strategies identified here with formal model transfer techniques (Barton et al. 2022) to further enhance long-term robustness and scalability of Raman-based monitoring models across bioprocess platforms.

## Supplementary Information

Supplementary Material

## Data Availability

The datasets generated and/or analyzed during the current study are not publicly available due to security regulations of GC Biopharma but are available from the corresponding author on reasonable request.
