# Supplementary material for: Variable-specific preprocessing enables cross-scale transferable Raman models from high-throughput bioreactor data
Source: Bioresour Bioprocess. 2026 Aug 29;13(1):122. doi: 10.1186/s40643-026-01105-5 (PMC13526000; doi:10.1186/s40643-026-01105-5)
Supplement: Supplementary file 1 [file 40643_2026_1105_MOESM1_ESM.docx]

**Supplementary Tables and Figures**

**Suppl. Table 1. Overview of experimental batches and coded operating conditions used in this study.** The table summarizes all 30 batches, including 23 Ambr® 250 batches and seven 5 L bench-scale bioreactor batches. The 23 Ambr® 250 batches served as the primary calibration dataset for the standard and variable-specific single-scale models. Of the seven bench-scale batches, two were used as additional calibration batches for the combined-scale models, whereas the remaining five were used as bench-scale prediction batches for cross-scale performance assessment and preprocessing pipeline comparison. Operating conditions are reported as coded relative levels to describe the experimental design structure. AM01–AM08 represent a full-factorial DoE designed to investigate the interaction between production temperature and pH. AM11–AM18 represent a full-factorial DoE designed to investigate the interaction between initial DO and production DO. AM09, AM10, and AM23 correspond to center-point conditions, while AM20–AM22 correspond to one-factor-at-a-time (OFAT) experimental conditions established for feed strategy development. Within each process parameter, identical coded levels indicate the same operating condition across batches. Values marked with an asterisk (*) indicate conditions outside the Ambr® 250 experimental range.

| Batch ID | Scale | Purpose | Production Temp | Initial  pH* | Production  pH | Initial  DO | Production  DO | Agitation Speed | Feed A | Feed B | Experimental design |
| --- | --- | --- | --- | --- | --- | --- | --- | --- | --- | --- | --- |
| AM01 | Ambr 250 | Calibration dataset | -1 | 0 | -1 | 0 | 0 | 0 | 0 | 0 | DOE |
| AM02 |  |  | -1 | 0 | 0 | 0 | 0 | 0 | 0 | 0 |  |
| AM03 |  |  | -1 | 0 | +1 | 0 | 0 | 0 | 0 | 0 |  |
| AM04 |  |  | 0 | 0 | -1 | 0 | 0 | 0 | 0 | 0 |  |
| AM05 |  |  | 0 | 0 | +1 | 0 | 0 | 0 | 0 | 0 |  |
| AM06 |  |  | +1 | 0 | -1 | 0 | 0 | 0 | 0 | 0 |  |
| AM07 |  |  | +1 | 0 | 0 | 0 | 0 | 0 | 0 | 0 |  |
| AM08 |  |  | +1 | 0 | +1 | 0 | 0 | 0 | 0 | 0 |  |
| AM09 |  |  | 0 | 0 | 0 | 0 | 0 | 0 | 0 | 0 | Center |
| AM10 |  |  | 0 | 0 | 0 | 0 | 0 | 0 | 0 | 0 |  |
| AM11 |  |  | 0 | 0 | 0 | -1 | -1 | 0 | 0 | 0 | DOE |
| AM12 |  |  | 0 | 0 | 0 | -1 | 0 | 0 | 0 | 0 |  |
| AM13 |  |  | 0 | 0 | 0 | -1 | +1 | 0 | 0 | 0 |  |
| AM14 |  |  | 0 | 0 | 0 | 0 | 0 | 0 | 0 | 0 |  |
| AM15 |  |  | 0 | 0 | 0 | 0 | +1 | 0 | 0 | 0 |  |
| AM16 |  |  | 0 | 0 | 0 | +1 | -1 | 0 | 0 | 0 |  |
| AM17 |  |  | 0 | 0 | 0 | +1 | 0 | 0 | 0 | 0 |  |
| AM18 |  |  | 0 | 0 | 0 | +1 | +1 | 0 | 0 | 0 |  |
| AM19 |  |  | 0 | 0 | 0 | 0 | 0 | +1 | 0 | 0 | OFAT |
| AM20 |  |  | 0 | 0 | 0 | 0 | 0 | 0 | -1 | 0 |  |
| AM21 |  |  | 0 | 0 | 0 | 0 | 0 | 0 | +1 | 0 |  |
| AM22 |  |  | 0 | 0 | 0 | 0 | 0 | 0 | 0 | +1 |  |
| AM23 |  |  | 0 | 0 | 0 | 0 | 0 | 0 | 0 | 0 | Center |
| BR01 | Bench-scale |  | +1 | 0 | 0 | 0 | 0 | 0 | 0 | 0 | N/A |
| BR02 |  |  | 0 | 0 | 0 | 0 | 0 | 0 | 0 | 0 |  |
| BR03 |  | Prediction dataset/  pipeline selection | 0 | 0 | 0 | 0 | 0 | -1 | 0 | 0 |  |
| BR04 |  |  | 0 | +1 | 0 | 0 | 0 | 0 | 0 | 0 |  |
| BR05 |  |  | 0 | +1 | 0 | 0 | 0 | -1 | 0 | 0 |  |
| BR06 |  |  | 0 | +1 | 0 | 0 | 0 | -1 | 0 | 0 |  |
| BR07 |  |  | +1 | +1 | 0 | 0 | 0 | 0 | 0 | 0 |  |

**Suppl. Table 2. Detailed performance metrics for standard single-scale and combined-scale Raman models.** Comprehensive overview of internal cross-validation and cross-scale prediction metrics, including latent variables (LV), R², Q², RMSEcv, RMSEP, and mean bias error of prediction (MBEP). The table contrasts the standard single-scale models with the combined-scale models across eight target outputs, quantifying the reduction in prediction errors and biases achieved through the combined modeling approach.

| Target  Outputs | Std.single | combined | Std.single | combined | Std.single | combined | Std.single | combined | Std.single | combined | Std.single | combined | Std.single | combined |
| --- | --- | --- | --- | --- | --- | --- | --- | --- | --- | --- | --- | --- | --- | --- |
|  | n | | LV | | R^2^ | | Q^2^ | | RMSEcv (%) | | RMSEP (%) | | MBEP | |
| VCD | 273 | 297 | 1+2+0 | 1+2+0 | 0.95 | 0.95 | 0.94 | 0.94 | 5.6 | 5.9 | 6.6 | 5.9 | -0.0245 | N/A |
| Viability | 273 | 294 | 1+2+0 | 1+3+0 | 0.75 | 0.80 | 0.70 | 0.76 | 6.6 | 4.7 | 15.8 | 4.4 | -0.1330 | N/A |
| Glucose | 273 | 297 | 1+2+0 | 1+3+0 | 0.98 | 0.98 | 0.98 | 0.97 | 4.0 | 4.2 | 5.3 | 4.9 | 0.0292 | 0.0301 |
| Lactate | 273 | 297 | 1+2+0 | 1+3+0 | 0.98 | 0.98 | 0.97 | 0.97 | 3.4 | 3.9 | 6.4 | 5.0 | -0.0001 | 0.0169 |
| Glutamine | 273 | 297 | 1+4+0 | 1+5+0 | 0.92 | 0.97 | 0.86 | 0.95 | 8.1 | 4.5 | 18.0 | 7.9 | -0.1618 | N/A |
| Glutamate | 273 | 297 | 1+3+0 | 1+4+0 | 0.97 | 0.97 | 0.96 | 0.95 | 4.3 | 4.5 | 13.5 | 8.4 | 0.1160 | -0.0612 |
| Ammonia | 273 | 297 | 1+3+0 | 1+4+0 | 0.96 | 0.96 | 0.95 | 0.95 | 6.0 | 6.1 | 10.7 | 8.9 | -0.0698 | -0.0407 |
| LDH | 218 | 235 | 1+2+0 | 1+2+0 | 0.91 | 0.91 | 0.89 | 0.90 | 5.9 | 5.4 | 10.7 | 5.8 | 0.0882 | -0.0381 |

**Suppl. Table 3. Comparison of 7-fold and leave-one-group-out (LOGO) cross-validation performance for four representative Ambr® 250 single-scale calibration models.** Q² and RMSEcv (%) values are shown for VCD, glucose, lactate, and glutamate using the standard and optimized preprocessing pipelines. LOGO cross-validation was performed using the 23 Ambr® 250 culture batches as groups to assess whether internal validation performance for these representative target outputs was substantially affected by within-batch correlations in the time-course calibration dataset.

| Metrics | Model | VCD | | Glucose | | | Lactate | | | Glutamate | |
| --- | --- | --- | --- | --- | --- | --- | --- | --- | --- | --- | --- |
|  |  | 7-fold | LOGO | 7-fold | LOGO | | 7-fold | LOGO | | 7-fold | LOGO |
| Q^2^ | Std. Single | 0.942 | 0.934 | 0.976 | | 0.972 | 0.975 | | 0.974 | 0.955 | 0.950 |
|  | Opt. Single | 0.939 | 0.933 | 0.975 | | 0.971 | 0.974 | | 0.973 | 0.954 | 0.950 |
| RMSEcv | Std. Single | 5.62 | 5.97 | 4.01 | | 4.29 | 3.42 | | 3.50 | 4.30 | 4.51 |
|  | Opt. Single | 5.76 | 6.04 | 4.11 | | 4.38 | 3.46 | | 3.53 | 4.37 | 4.56 |

**Suppl. Table 4.** **Quantitative comparison of cross-scale prediction performance among the standard single-scale, optimized single-scale, and combined-scale Raman models.** RMSEP reduction (%) was calculated relative to the standard single-scale model as [(RMSEPstd.–RMSEPopt,)/ RMSEPstd.]×100. Gap closure versus the combined-scale benchmark (%) was calculated as [(RMSEPstd.−RMSEPopt.)/(RMSEPstd−RMSEPcombined)] ×100. A value of 100% indicates that the optimized single-scale model achieved the same RMSEP as the combined-scale benchmark; values between 0% and 100% indicate partial gap closure, and values greater than 100% indicate performance exceeding the combined-scale benchmark.

| Target  Outputs | Std. Single  RMSEP(%) | Opt. Single  RMSEP(%) | Combined-scale  RMSEP(%) | RMSEP reduction  vs. Std. (%) | Gap closure  vs. Combined (%) |
| --- | --- | --- | --- | --- | --- |
| Viability | 15.8 | 10.0 | 4.4 | 36.7 | 50.9 |
| Glutamine | 18.0 | 11.7 | 7.9 | 35.0 | 62.4 |
| Glutamate | 13.5 | 7.3 | 8.4 | 45.9 | 121.6 |
| Ammonia | 10.7 | 9.2 | 8.9 | 14.0 | 83.3 |
| LDH | 10.7 | 4.7 | 5.8 | 56.1 | 122.4 |

**Suppl. Figure 1. Workflow of the standardized Raman spectral data preprocessing and quality control.** Step-by-step schematic of the preprocessing workflow applied to the raw Raman spectra. Prior to baseline correction and normalization, all acquired spectra underwent a standardized quality control procedure to identify and remove cosmic spikes and other pronounced spectral artifacts that are unrelated to chemical composition.

**Suppl. Figure 2.** **Heatmap-based comparison of VIP-derived regions and coefficient patterns within VIP > 1 Raman shift regions for representative target outputs.** (A) Heatmap of Raman shift regions with VIP scores greater than 1 for glucose, lactate, glutamate, and VCD. High-VIP regions were broadly shared among the target outputs, indicating that VIP primarily captured common process-related spectral variance in the cell culture dataset. (B) Heatmap of model coefficient values within the VIP > 1 Raman shift regions. In contrast to the VIP-based pattern, coefficient values within these regions showed more target-dependent distributions, supporting the use of coefficient-based analysis to further interpret Raman regions contributing to target-specific model predictions.

**Suppl. Figure 3.** **Raw Raman spectra from Ambr® 250 and bench-scale bioreactors.** Raw Raman spectra acquired from the Ambr® 250 high-throughput system (blue lines) and the bench-scale bioreactors (green lines). The spectral overlay reveals distinct differences in baseline levels, fluorescence background, and overall signal intensity between the two systems. These variations represent the cross-condition challenge inherently present during model transfer, underscoring the necessity for robust spectral preprocessing.
